# Utility of Whole-Genome Sequencing of Escherichia coli O157 for Outbreak Detection and Epidemiological Surveillance

**DOI:** 10.1128/JCM.01066-15

**Published:** 2015-10-16

**Authors:** Anne Holmes, Lesley Allison, Melissa Ward, Timothy J. Dallman, Richard Clark, Angie Fawkes, Lee Murphy, Mary Hanson

**Affiliations:** a Scottish *E. coli* O157/VTEC Reference Laboratory (SERL), Royal Infirmary of Edinburgh, Edinburgh, Scotland; b Wellcome Trust Clinical Research Facility (WTCRF), University of Edinburgh, Western General Hospital, Edinburgh, Scotland; c Centre for Immunity, Infection and Evolution, School of Biological Sciences, University of Edinburgh, Edinburgh, Scotland; d Gastrointestinal Bacterial Reference Unit, Microbial Services Division, Public Health England, London, United Kingdom

## Abstract

Detailed laboratory characterization of Escherichia coli O157 is essential to inform epidemiological investigations. This study assessed the utility of whole-genome sequencing (WGS) for outbreak detection and epidemiological surveillance of E. coli O157, and the data were used to identify discernible associations between genotypes and clinical outcomes. One hundred five E. coli O157 strains isolated over a 5-year period from human fecal samples in Lothian, Scotland, were sequenced with the Ion Torrent Personal Genome Machine. A total of 8,721 variable sites in the core genome were identified among the 105 isolates; 47% of the single nucleotide polymorphisms (SNPs) were attributable to six “atypical” E. coli O157 strains and included recombinant regions. Phylogenetic analyses showed that WGS correlated well with the epidemiological data. Epidemiological links existed between cases whose isolates differed by three or fewer SNPs. WGS also correlated well with multilocus variable-number tandem repeat analysis (MLVA) typing data, with only three discordant results observed, all among isolates from cases not known to be epidemiologically related. WGS produced a better-supported, higher-resolution phylogeny than MLVA, confirming that the method is more suitable for epidemiological surveillance of E. coli O157. A combination of *in* silico analyses (VirulenceFinder, ResFinder, and local BLAST searches) were used to determine *stx* subtypes, multilocus sequence types (15 loci), and the presence of virulence and acquired antimicrobial resistance genes. There was a high level of correlation between the WGS data and our routine typing methods, although some discordant results were observed, mostly related to the limitation of short sequence read assembly. The data were used to identify sublineages and clades of E. coli O157, and when they were correlated with the clinical outcome data, they showed that one clade, Ic3, was significantly associated with severe disease. Together, the results show that WGS data can provide higher resolution of the relationships between E. coli O157 isolates than that provided by MLVA. The method has the potential to streamline the laboratory workflow and provide detailed information for the clinical management of patients and public health interventions.

## INTRODUCTION

Shiga toxin-producing Escherichia coli (STEC) strains are important gastrointestinal pathogens and common causes of acute renal failure in children worldwide (1, 2, 3). In Scotland, the epidemiology and clinical outcome of E. coli O157 infection in humans has been closely monitored by Health Protection Scotland (HPS) since the introduction of enhanced surveillance in 1999, following a rapid increase in the number of microbiologically confirmed cases of STEC infection during the mid-1990s. Enhanced surveillance was extended in 2003 to include non-O157 STEC, and enhanced surveillance of hemolytic-uremic syndrome (HUS) was established in 2003. The most common STEC serogroup isolated from patients in Scotland is E. coli O157. In the past 5 years, the number of culture-confirmed cases of E. coli O157 infection in Scotland ranged from 195 to 263 per year, compared with 22 to 75 cases of non-O157 STEC infection per year (4, 5). On average, 43% of the STEC infection cases in Scotland require hospitalization and 9% progress to HUS, mostly in children (5). The reported rate of E. coli O157 infection in Scotland (4.9 cases per 100,000 population in 2014 [HPS personal communication]) is higher than in most countries, including other countries within the United Kingdom (5, 6). The reasons for this are unclear and likely to be multifactorial but may be related to the relative densities of human and cattle populations, which are the main reservoir of E. coli O157. Although large outbreaks of infections associated with food products have been reported in Scotland (7, 8, 9), the majority of infections are apparently sporadic, and contact with animal feces, exposure to farm animals or farm environments, and drinking of water from private water supplies have all been identified as strong risk factors for infection (10, 11).

The continued use of appropriate control measures, which is dependent on the prompt diagnosis of cases and detection of outbreaks, is essential to limit the spread of this pathogen. The Scottish Escherichia coli O157/Verotoxigenic E. coli Reference Laboratory (SERL) works closely with Scottish National Health Service (NHS) boards and with HPS and plays a pivotal role in case confirmation and outbreak detection by providing identification and typing services for E. coli O157 and other STEC isolates. Putative isolates of E. coli O157 are referred to the SERL from all 14 Scottish health boards for confirmation of identity and typing. In addition, fecal samples from cases with typical symptoms of STEC, but culture negative in local diagnostic laboratories, are sent to the SERL for further analysis. Currently the SERL uses a range of phenotypic and genotypic typing techniques, including real-time-PCR, phage typing, disc diffusion susceptibility testing, multilocus variable-number tandem-repeat analysis (MLVA), and pulsed-field gel electrophoresis (PFGE). However, there is an ever-increasing body of evidence demonstrating the potential benefits of WGS as a tool that may replace these methods (12, 13, 14, 15, 16). Bacterial WGS is becoming increasingly affordable and has demonstrated better resolution than more conventional typing methods that sample only a fraction of the genome (17, 18, 19). As a reference laboratory, it is essential that we continually aim to improve our services to provide precise and timely data to support public health interventions. In this study, we have compared our current methods with WGS for the typing and characterization of E. coli O157 for epidemiological surveillance and detection of outbreaks. In addition, we have correlated genome content with pathogenicity and ancestry to try to achieve a better understanding of the genetic factors associated with virulence in STEC.

## MATERIALS AND METHODS

### Bacterial isolates.

Isolates of E. coli O157 (*n* = 105) from human fecal samples received in Lothian laboratories between 1 April 2007 and 31 March 2012 were analyzed in this study (see Table S1 in the supplemental material). These included isolates from 10 cases received over an 11-month period in 2011 associated with a United Kingdom-wide outbreak that was linked to unwashed vegetables (20), isolates that were epidemiologically linked by place and time (eight single-household clusters, one cluster involving two households that was farm related, and one travel-associated cluster), and sporadic isolates from 27 patients thought to have acquired their infections outside the United Kingdom. The patients had all been interviewed by using standardized questionnaires to collect information, including severity of infection and travel history, to identify any epidemiological links and try to pinpoint the source of infection. The ages of the patients sampled ranged from 1 to 85 years, with a gender distribution of 42% male and 58% female.

### DNA isolation.

E. coli O157 isolates were incubated overnight at 37°C on sorbitol MacConkey agar (Oxoid Ltd., Basingstoke, United Kingdom). DNA was extracted with the Wizard Genomic DNA purification kit (Promega Ltd. UK, Southampton, United Kingdom) as described by the manufacturer. The quality of the genomic DNA (gDNA) was checked by gel electrophoresis, and the quantity and purity were measured with the NanoDrop 1000 apparatus (NanoDrop Products, Thermo Scientific).

### Phenotypic testing.

Phage typing was performed with 16 phages as previously described (21). β-Glucuronidase (β-GUD) production was assessed by using TBX agar (tryptone bile X-glucuronide agar; E&O Laboratories); ATCC 25922 was used as a control strain. Antibiotic sensitivity patterns were determined by the disc diffusion method with 15 antibiotics routinely tested in the SERL for surveillance purposes: chloramphenicol, ciprofloxacin, ampicillin, gentamicin, streptomycin, meropenem, nalidixic acid, kanamycin, tetracycline, trimethoprim, piperacillin-tazobactam, cefotaxime, ceftazidime, co-amoxiclav and co-trimoxazole. The European Committee on Antibiotic Susceptibility Testing (EUCAST) criteria were used to determine resistance.

### MLVA.

MLVA was performed as previously described (22). Raw data (.fsa files) from an ABI 3130 genetic analyzer (Applied Biosystems) were imported and analyzed in BioNumerics v6.6 (Applied Maths, Sint-Martens-Latem, Belgium) with the MLVA plugin. A minimum spanning tree was produced with the MLVA allele numbers.

### Shiga toxin gene subtyping.

PCR assays and gel electrophoresis were performed as described by Scheutz et al. (23), with a few modifications. A sample volume of 15 μl with a HotStarTaq master mix kit (Qiagen UK Ltd., Crawley, United Kingdom), 0.2 μM primer(s), and 1.5 μl of DNA was used. *stx*_1a_, *stx*_1c_, and *stx*_1d_ primers were multiplexed in a single reaction mixture. *stx*_2_ primers for *stx*_2b_, *stx*_2e_, and *stx*_2g_ were multiplexed, while *stx*_2a_, *stx*_2c_, *stx*_2d_, and *stx*_2f_ were used in singleplex reaction mixtures. The thermocycler conditions were 95°C for 15 min and then 35 cycles of 94°C for 50 s, 66°C for 40 s, and 72°C for 3 min. Amplicons were run on a 2% agarose gel.

### WGS.

WGS was performed with the Ion Torrent Personal Genome Machine (PGM; Life Technologies, Carlsbad, CA) at the Wellcome Trust Clinical Research Facility, Edinburgh, Scotland. Samples were sequenced retrospectively over a period of ∼1 year on the same machine by the same personnel and analyzed with the same version of the software to limit the effects of batching. Libraries were generated with 1 μg of the gDNA and enzyme fragmented with the Ion XpressPlus Fragment Library kit. Specifically, gDNA was fragmented into 200- to 300-bp blunt-ended DNA fragments. The fragmented DNA was ligated to Ion Torrent-compatible barcoded adapters; this was followed by nick repair to complete the linkage between the adapters and DNA inserts. The adapter-ligated library was then size selected for optimum length (330 bp), and the final library was amplified. An aliquot of the library was analyzed on the Bioanalyzer instrument with an Agilent High Sensitivity DNA kit to assess the size distribution and determine the molar library concentration. Two barcoded libraries were pooled at a concentration of 100 pM, and a 10 pM portion of the pool was added into an emulsion PCR-based template reaction mixture; in this reaction mixture, the fragments generated during library preparation were attached to Ion Sphere particles (ISPs) and clonally amplified. This process was carried out with the Ion One Touch 2 system and the Ion PGM Template OT2 200 kit. Quality control was performed on the Qubit 2.0 fluorometer with the Ion Sphere Quality Control assay. The optimal amount of library corresponds to the library dilution that gives template ISP percentages of 10 to 30%. The template-positive ISPs were then enriched and sequenced on the PGM with the Ion PGM Sequencing 200 kit v2 and an Ion 316 chip. Average coverage was determined by using an estimated genome size of 5.528 Mb and found to be 42× (range, 23 to 106×; see Table S1 in the supplemental material).

### Bioinformatics.

Sequence reads were mapped to the reference strain (Sakai, GenBank accession no. NC_002695) with BWA-MEM (24), and isolates with an average coverage of <20× were excluded from the analysis. Single nucleotide polymorphisms (SNPs) were then identified with GATK2 (25) in unified genotyper mode. Core genome positions (defined as sites for which a base was called for all isolates) that had a high-quality SNP (>90% consensus, minimum depth of 10×, genotype quality score of ≥30) or were the same as the reference base were extracted to produce a whole-core genome alignment for phylogenetic analysis. Sequence reads were also assembled *de* *novo* with the Torrent Suite Software, and the assemblies (contigs) were used for *in* silico analysis as described below.

### *In* silico analysis.

Local BLAST databases were developed in either BioEdit (http://www.mbio.ncsu.edu/bioedit/bioedit.html) or BioNumerics v6.6 (Applied Maths) for multilocus sequence typing (MLST), *stx* subtyping, virulence gene detection, and lineage-specific polymorphism assay (LSPA-6) typing. The databases were then queried with the *de* *novo* assemblies. For MLST, the database consisted of the alleles of 15 housekeeping genes (*arcA*, *aroE*, *aspC*, *clpX*, *cyaA*, *dnaG*, *fadD*, *grpE*, *icdA*, *lysP*, *mdh*, *mtlD*, *mutS*, *rpoS*, *uidA*), which were downloaded from the EcMLST website (www.shigatox.net/ecmlst/cgi-bin/index); for *stx* subtyping, the database consisted of 600-bp sequences (342 bp of the C-terminal part of subunit A and 258 bp of the N-terminal part of subunit B) of 106 *stx* variants used by Scheutz et al. (23). For LSPA-6 typing, partial gene sequences of the six genes (*folD-sfmA*, *Z5935*, *yhcG*, *rbsB*, *rtcB*, and *arp-iclR*) were extracted from strain Sakai and used to form the database (26, 27). Isolates with genotype 111111 were classified as LSPA-6 lineage I, those with genotype 211111 were classified as LSPA-6 lineage I/II, and those with other derivations were classified as LSPA-6 lineage II.

Strains belonging to clade 8 (28) were identified on the basis of the discriminatory SNP (C/A at position 539 in gene ECs2357) described by Riordan et al. (29).

VirulenceFinder and ResFinder (http://cge.cbs.dtu.dk/services/) were used to determine the presence of E. coli virulence genes and acquired antibiotic resistance genes with identity thresholds of 85 and 98%, respectively (30, 31).

### Data analysis.

Ridom EpiCompare (http://www3.ridom.de/epicompare/) was used to calculate the discriminatory power of the typing methods. Odds ratios (ORs) were calculated with MedCalc (http://www.medcalc.org/calc/odds_ratio.php).

### Recombination detection.

The number of variable sites across the core genome alignment was calculated in sliding windows of 10,000 bp and plotted by using custom Python and R scripts to identify areas of the genome with an unusually high density of SNPs. The entire E. coli O157 core genome alignment was screened for recombination with BratNextGen (32) and also screened for recombination with BratNextGen when the two highly divergent and putative recombinant sequences (XH18570E and XH22083W) were excluded from the alignment.

### Maximum-likelihood phylogenetic analysis.

Maximum-likelihood core genome phylogenies were constructed with the RaxML software (33; Linux version 8) and PhyML (34). For the published RaxML phylogeny, the general time-reversible model of nucleotide substitution was used, with gamma-distributed rate heterogeneity across sites and 1,000 bootstrap replicates. Neighbor-joining phylogenies were also produced with MEGA version 5 (35). We confirmed that the tree topology was robust to the use of different software frameworks, as well as different choices of evolutionary model. In the absence of an obvious outgroup to O157, phylogenies were rooted at the midpoint between the two most divergent taxa in the trees.

### Nucleotide sequence accession numbers.

FASTQ sequences were deposited in the NCBI Sequence Read Archive under BioProject no. PRJNA283577 (http://www.ncbi.nlm.nih.gov/bioproject/PRJNA283577/).

## RESULTS

### Analysis of core genome.

Our core genome alignment of the 105 E. coli O157 strains was 4,122,236 bp in length and contained 8,721 variable sites. However, a large number (4,110; 47%) of the SNPs were attributable to six “atypical” E. coli O157 strains, including a sorbitol-fermenting (SF) E. coli O157 strain (XH25052C) and three β-GUD-positive E. coli O157 strains (XH20443W, XH16200L, and XH24967A). When looking across the whole alignment, an ∼150-kb region with a high density of SNPs (an average of 148.33 variable sites per 10,000-bp window, compared to a genome-wide average of 21.16 variable sites per 10,000-bp window) was observed near the end of the genome. Two of the atypical strains (XH18570E and XH22083W) were identified by the BratNextGen software as recombinant in this region, suggesting that genetic material has entered from a donor strain. See Fig. S1 in the supplemental material for plots of the distribution of variable sites across the genome in the presence and absence of recombinant strains XH18570E and XH22083W, as well as with and without recombinant regions excluded. Sanger sequencing of 792 bp of the *mutL* gene, which lies within the putative recombinant region, confirmed that the SNPs detected by WGS were genuine, and a BLAST analysis of this gene region showed that it was identical to the *mutL* gene of E. coli K-12.

Removal of recombinant strains XH18570E and XH22083W from the alignment, followed by the removal of additional, small, putative recombinant regions identified by BratNextGen for other strains, resulted in a 4,114,451-bp alignment containing 6,626 variable sites.

### Epidemiological concordance.

**FIG 1:**
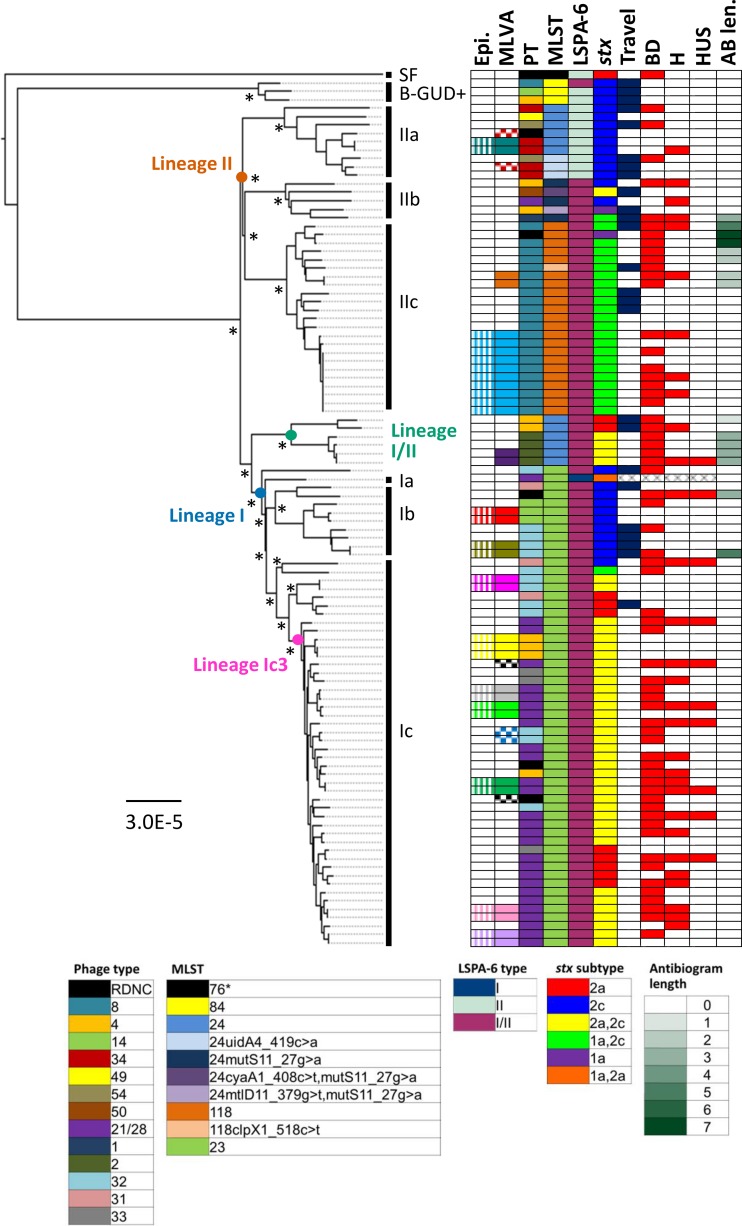
Maximum-likelihood core genome phylogeny for 103 E. coli O157 isolates from Lothian, Scotland. Recombinant sequences XH18570E and XH22083W were excluded. The tree was constructed with RAxML by using a general time-reversible model of nucleotide substitution and gamma distributed rate heterogeneity across sites. Branch lengths are in numbers of substitutions per site. Isolates known to be epidemiologically related (Epi) are in the same color. MLVA types shared by more than one isolate are indicated by filled boxes of the same color; SLV MLVA types are indicated by checkered boxes of the same color. PTs, multilocus STs, LSPA-6 types, *stx* subtypes, and the number of antibiotics (out of 15 tested) to which an isolate was resistant (AB len.) are shown. RDNC indicates that the PT reaction did not conform to a recognized pattern. Isolates associated with recent travel are in blue (Travel). All Lothian isolates were associated with diarrhea; isolates associated with bloody diarrhea (BD), hospitalization (H), and/or HUS are in red. Sublineages, defined as described in the text, are indicated by vertical black bars. One thousand bootstrap replicates were conducted, and bootstrap values of >98%, for nodes corresponding to the major lineages and sublineages described in the text, are indicated by asterisks.

Eleven groups of two or more isolates known to be epidemiologically linked were present in our analysis, and the epidemiologically linked isolates formed clusters with 100% bootstrap support in the core genome phylogeny (Fig. 1). Within each epidemiologically linked cluster, there were fewer than four core SNPs separating the isolates. Three SNPs were identified among 10 isolates from a United Kingdom-wide phage type 8 (PT8) outbreak spanning 11 months in 2011. A maximum of two SNPs were identified among isolates from the other epidemiologically linked cases received by the SERL within a shorter time frame (0 to 14 days). The SNPs detected among epidemiologically related cases were confirmed by Sanger sequencing. For the SNP distances between all of the pairs of isolates, see Table S2 in the supplemental material. In three instances, isolates from cases with no apparent epidemiological link clustered together in the core SNP phylogeny and were separated by a maximum of 1 SNP. In two cases (XH11963F with XH12059K and XH15449Q with XH15521V), the isolates were temporally related (isolated within 8 days), had the same MLVA profile, and did not differ at any core SNP positions (i.e., their core genome sequences were identical), suggesting that there may have been an unidentified common source of infection. Another isolate (XH16734G) clustered with XH15449Q and XH15521V (differing from those isolates by one SNP) but was an MLVA double-locus variant (DLV) and was isolated ∼4 months later. In the third case (XH11856D with XH12193B), the two isolates differed by one SNP and clustered in the core genome phylogeny with 100% bootstrap support, were MLVA single-locus variants (SLVs), and were isolated 39 days apart. Two additional MLVA SLVs (XH14013B with XH17884G and XH14653V with XH18908N) were observed in the data set; however, they were not related in time or space and differed by 36 and 126 SNPs, indicating that they were not closely related. The genetic variation among all pairs of isolates with no epidemiological links was 9 to 1,632 SNPs (see Table S2 in the supplemental material).

### Concordance with MLVA and phage typing.

A comparison of the minimum spanning tree produced by MLVA and the phylogeny based on the core genome showed that, although epidemiologically linked isolates clustered together in both trees, the overall topologies and the clustering of epidemiologically unrelated isolates differed between the trees. Within the core genome phylogeny, all of the sublineages we defined, and the majority of the clades within these were supported by bootstrap values of 100% (see Fig. S2 and S3 in the supplemental material). In contrast, the minimum spanning tree based upon the MLVA data (see Fig. S4 in the supplemental material) had relatively low bootstrap values, suggesting that MLVA provides meaningful phylogenetic relationships only for closely related isolates. Similarly, although phage typing was broadly congruent with the core genome phylogeny, identical PTs were distributed across the core genome phylogeny in different clades (Fig. 1). For example, PT4 was distributed in four of our defined sublineages across the tree, suggesting a lack of specificity of phage typing.

### Discriminatory power of typing methods.

The SNP data separated the 105 isolates into 81 different types (Simpson's index of diversity [SID], 0.989; 95% confidence interval [CI], 0.979 to 0.998), where isolates that differed by more than three SNPs were considered to be of different types. MLVA produced 80 different allelic profiles (SLVs were considered to be of the same type) and had the same discriminatory index as the SNP method (SID, 0.989; 95% CI, 0.979 to 0.998). The types were concordant, except for the two sets of MLVA SLV's that fell in different parts of the core genome phylogeny and isolate XH16734G, which differed at two MLVA alleles (VNTR3 and VNTR9 by one repeat) from XH15449Q and XH15221V (Fig. 1; see Table S1 in the supplemental material). Phage typing produced 14 different lysis patterns and had a much lower SID than the other two methods (SID, 0.853; 95% CI, 0.817 to 0.89).

### *stx* subtyping.

The *stx* subtypes were determined by PCR and *in* silico analyses. A relatively low diversity of subtypes was detected among the 105 isolates (Fig. 1; see Table S1 in the supplemental material). The most common subtype was *stx*_2a_/*stx*_2c_ (*n* = 42), followed by *stx*_2c_ only (*n* = 26) and *stx*_1a_/*stx*_2c_ (*n* = 24). Eleven isolates carried *stx*_2a_ only, while two had *stx*_1a_ only.

The PCR results were concordant with the local BLAST and VirulenceFinder results for 102 (97%) and 97 (92%) isolates, respectively. The local BLAST search did not detect the *stx*_2c_ gene in three isolates found to carry *stx*_2a_ and *stx*_2c_ by PCR; in one case, only a partial sequence was identified, while in another, two different *stx*_2a_ subtypes were detected. VirulenceFinder produced similar results for these isolates; however, in one case, it detected the presence of an *stx*_2c_ subunit A but the adjacent subunit B was reported as *stx*_2a_. Similarly, in four other cases, VirulenceFinder detected different *stx*_2_ variants in subunits that lay beside each other in the genome. In one case, a *stx*_2d_ subunit A was reported in one isolate, which was not detected by PCR assay or the local BLAST search.

### Virulence gene detection.

VirulenceFinder and local BLAST searches were used to determine the presence of virulence genes; for a complete list of the genes detected in each isolate, see Table S1 in the supplemental material. A total of 23 virulence genes were detected among the 105 isolates. Twenty of the genes (*astA*, *eae*, *ehxA*, *espA*, *espB*, *espF*, *espJ*, *espP*, *etpD*, *gad*, *iha*, *iss*, *katP*, *nleA*, *nleB*, *nleC*, *prfB*, *tccP*, *tir*, and *toxB*) were present or partially present (<85% of the gene detected; see Table S1 in the supplemental material) in the majority (94%) of the isolates. In six isolates, one or more of these genes were not detected (see Table S1). These were the four “atypical” E. coli isolates (XH16200C, XH20443W, XH24967A, and XH25052C), one isolate that was negative for *espP* (XH12849W), and another that was negative for *iha* (XH14120C). The three remaining genes, *cdtB*, *cba*, and *celB*, were found in only a small proportion of the isolates.

The presence of *eae* and *hly* was detected by PCR in all of the study isolates; however, VirulenceFinder failed to detect these genes in 8 and 10 cases, respectively (see Table S1). The discordant results were further analyzed by performing local BLAST searches of these genes against the *de* *novo* assemblies. These revealed that the genes were present in all of the strains but were split among two or more different contigs, which explains why they were not detected by VirulenceFinder. Also, in some cases, part of the gene sequence was duplicated in the different contigs. For example, for XH11963F, bases 1 to 1602 of *eae* were present in contig c122 and bases 1388 to 2805 were present in contig c131, resulting in an overlap of 125 bp in the middle of the gene. The fractured and duplicated nature of these assemblies may be suggestive of low coverage in these areas, resulting in poor assembly.

Ten other genes not tested by PCR (*espF*, *espP*, *nleC*, *iha*, *katP*, *toxB*, *tccP*, *cba*, *celB*, *cdtB*) were further investigated by local BLAST searches, as they were not detected by VirulenceFinder in at least one of the isolates. The results of the local BLAST searches matched the VirulenceFinder results in the majority of the cases (313/413), where the genes were also not detected by the local BLAST searches. In 10 cases, only partial genes (<85% of the gene) were detected, while in 90 cases, the genes were present in multiple contigs, explaining why they were not detected by VirulenceFinder. Most notably, *tccP* was not detected in 75/105 cases because of its presence in multiple contigs (see Table S1 in the supplemental material). *tccP* can vary in length and contains proline-rich repeats (36), which are difficult to resolve by using short sequence reads, explaining its presence in multiple contigs and the failure of VirulenceFinder to detect this gene in most of the isolates.

### Antimicrobial resistance gene detection.

The isolates were tested for susceptibility to 15 different antimicrobial agents belonging to six different antimicrobial classes. A relatively low level of resistance was detected (Fig. 1; see Table S1 in the supplemental material); 15 (14%) out of 105 isolates showed resistance to one or more antibiotics. Streptomycin (*n* = 14), sulfamethoxazole (*n* = 14), and tetracycline (*n* = 9) were the antibiotics to which resistance was most frequently observed. Two isolates were resistant to six antibiotics: ampicillin, kanamycin, sulfamethoxazole, streptomycin, tetracycline, and trimethoprim. We did not observe an association between antimicrobial resistance and foreign travel (OR, 1.2; *P* = 0.7753). There were no disagreements between the observed and predicted susceptibility patterns; however, phenotypic and genotypic resistance to several antibiotics (ampicillin, sulfamethoxazole, streptomycin, trimethoprim-sulfamethoxazole, and trimethoprim) was observed in one isolate that was epidemiologically linked to a strain in which no resistance was detected (XH18778Q and XH18795X; see Table S1 in the supplemental material). This may be related to the loss or gain of an unstable plasmid carrying the resistance genes.

### MLST.

Ten different multilocus sequence types (STs) were identified among the 105 strains (Fig. 1). ST23 was the most common type (*n* = 56), followed by ST118 (*n* = 22) and ST24 (*n* = 14). The three β-GUD-positive strains belonged to ST84 (ST65 seven-locus scheme), while the SF E. coli O157 strain was ST76, which corresponds to previous reports (37). Five new STs were identified, as they differed by at least one SNP, in one or more alleles, from those present in the EcMLST database. Also, in four strains, a single nucleotide gap/no call was detected in one of the alleles (see Table S1 in the supplemental material). Inspection of the sequence reads at these positions showed that the base was present in only about one-third of the reads and so would have been excluded during minimum variant frequency filtering. As expected, the ST correlated well with the core genome phylogeny.

### Evolutionary analysis of E. coli O157.

Figure 1 shows the maximum-likelihood phylogeny constructed from the core genome alignment of the O157 strains. The atypical strains (SF and β-GUD positive) cluster together and are phylogenetically distinct from the β-GUD-negative E. coli O157 strains isolated from the majority of Lothian patients in recent years. The rest of the phylogeny can be divided into three clades that correspond to recently described lineages I, I/II, and II (38). As noted by Dallman et al., LSPA-6 typing does not adequately resolve these lineages; the *folD-sfmA* polymorphism does not differentiate lineages I and II. The lineages can be further divided into sublineages with distinct genotypic and phenotypic characteristics. As indicated in Fig. 1, the bootstrap values for all of the lineages and sublineages were greater than 98% (with the majority being 100%), indicating a high level of support for the clusters observed. A strong concordance was noted between the lineages defined in the phylogeny and the clusters inferred from the proportion of shared ancestry tree in the BratNextGen analysis (see Fig. S2 in the supplemental material). Sublineage IIa consists of ST24 strains, or ST24 SLVs, that carry *stx*_2c_ only and consist of PT34, PT49, and PT54. Sublineage IIb is a heterogeneous group with respect to the PT and *stx* profile, while sublineage IIc consists of ST118 PT8 strains that have acquired *stx*_1a_. Fifty-seven percent of the strains associated with antimicrobial resistance (antibiogram length of >1) were present within lineage IIc. Lineage I/II consists of ST24 PT4 and PT2 strains carrying either *stx*_2a_ only or both *stx*_2a_ and *stx*_2c_, respectively, and belonging to clade 8 (see Table S1 in the supplemental material) that have been associated with large outbreaks of severe disease in the United States (28). Lineage I strains belong to ST23 and consist of three main sublineages (Ia, Sakai; Ib, *stx*_2c_-only genotype; and Ic) that can be further subdivided into three main clades. The largest, named Ic3 here, consists predominately of PT21/28 *stx*_2a_ and *stx*_2c_ strains that are responsible for the majority of the human infections reported in Scotland and the United Kingdom (5). Of note, no isolates in this clade were associated with acquired antimicrobial resistance or foreign travel.

### Correlation of genotype with disease severity.

The enhanced surveillance data showed that all 105 (100%) patients had diarrhea, 64 (61%) had bloody diarrhea, 30 (29%) were hospitalized, and 10 (9%) developed HUS. Six (60%) of the 10 HUS cases were in children <16 years old (see Table S1 in the supplemental material). No deaths were recorded.

Seven (70%) of the 10 HUS cases were associated with genotype Ic3. Statistical analysis of the results showed a significant association between Ic3 and HUS (OR, 4.5938; *P* = 0.0351). Ic3 was also significantly associated with hospitalization (OR, 3.1503; *P* = 0.0103) but not bloody diarrhea (OR, 2.1212; *P* = 0.0826). No significant correlations between the other genotypes and clinical outcomes were observed; however, some tests were based upon very small numbers of samples, making it difficult to determine statistically meaningful results.

Ic3 strains carry *stx*_2a_ (and usually *stx*_2c_), and previous reports have suggested that *stx*_2a_ is associated with increased virulence and more severe human disease (39, 40). We found that 8 (80%) of 10 isolates from HUS cases carried the *stx*_2a_ gene; however, statistical analysis showed that the presence of *stx*_2a_ was not significantly associated with HUS (OR, 4.4444; *P* = 0.0678) or hospitalization (OR, 2.0829; *P* = 0.0986), suggesting that other features of 1c3 and/or host factors are likely to be important in the development of severe complications of infection.

## DISCUSSION

Two of the most important features of a typing method are its ability to distinguish between unrelated strains and to correctly classify epidemiologically related isolates from an outbreak or cluster as part of the same clone (41). This study showed that the identification of core genome SNPs following WGS provided a highly discriminatory method for subtyping of E. coli O157, giving results concordant with the epidemiological data. The method was also concordant with MLVA for the identification of epidemiologically linked cases, even though different areas and a much smaller percentage of the genome were targeted by MLVA, demonstrating the value of MLVA for outbreak detection. A recent study carried out by Public Health England (PHE) showed that MLVA was as sensitive as WGS for identifying linked cases of E. coli O157 infection but, because of the time taken to determine the relatedness of MLVA DLVs occasionally observed in larger outbreaks, found that WGS resolved all of the cases in a cluster faster (42). In this study, among the isolates with no known epidemiological links, there were three discordant results between MLVA and WGS. In one case, an MLVA DLV was shown to differ by only one SNP from two other isolates (that were identical by WGS and MLVA), while in two cases, the WGS data showed that MLVA SLVs were not, in fact, closely related in terms of the core genome. We currently use MLVA profiles (exact matches and SLVs) to alert health protection teams and HPS of putative linked cases, which are then further investigated for epidemiological links. Together, the data suggest that WGS may provide more precise data and reduce the unnecessary deployment of health protection resources in the investigation of putatively linked isolates. Turabelidze et al. (14) showed that a cluster of E. coli O157 cases associated with the consumption of romaine lettuce and salad bar exposures was better resolved by using core genome SNPs than by using MLVA and PFGE.

The determination of SNP distances between strains showed that some diversity existed among isolates from epidemiologically linked cases. We found three or fewer SNP differences among isolates from known epidemiologically linked cases. Definition of variation is important for the assessment of relatedness and establishment of a targeted approach to case inclusion in outbreaks and contact tracing. Others have reported SNP diversity among epidemiologically related cases. Dallman et al. (42) found five or fewer SNP differences among 183 epidemiologically related isolates from different clusters received by PHE, Underwood et al. (17) detected four SNP differences among 16 isolates of E. coli O157 isolated during a farm outbreak, and Joensen et al. detected seven SNP differences among six outbreak cases (30). Various factors may explain the degree of genomic variation reported in different outbreaks, including the strains sampled, outbreak duration, the sequencing platforms used, and the criteria used to define high-quality SNPs.

In the past, considerable effort has been made to standardize E. coli typing methods to enable comparisons of data produced in different laboratories (e.g., PulseNet, CDC). The standardization of MLVA between reference laboratories in the United Kingdom has been invaluable for the rapid detection of cross-border outbreaks (22), and this study has confirmed its utility in outbreak detection. It is likely that the standardization of data produced by WGS in different laboratories will be a major challenge and require considerable collaboration between clinical and reference laboratories.

A major advantage of WGS is its ability to produce a well-supported, high-resolution phylogeny and therefore an appropriate method for understanding or tracking the evolutionary relationships between strains and for detailed epidemiological surveillance of E. coli O157. Although the isolates sequenced in this study were from a single Scottish health board, phylogenetic analysis revealed a population structure remarkably consistent with that in previous studies, in particular, work recently carried out at PHE, where a large number of strains of E. coli O157 (*n* = 1,075) from clinical and animal sources collected over a 29-year period in the United Kingdom were analyzed (38). All lineages and sublineages, except Ia, were represented in our strain collection. Similar to the PHE study, the main clusters within lineages I, I/II, and II consisted of isolates belonging to the PTs commonly associated with human disease in the United Kingdom, PT21/28, PT2, and PT8, respectively.

Another major advantage of WGS is the additional information that can be extracted, including important virulence and antibiotic resistance determinants. We were able to correctly infer antibiotic susceptibility profiles from the WGS data, suggesting that WGS is a suitable alternative to current routine laboratory testing for the surveillance of antimicrobial resistance and the detection of emerging resistance phenotypes. Resistance to streptomycin, sulfamethoxazole, and tetracycline was most often observed, consistent with previous reports (43, 44, 45), most likely because of the selection pressure imposed by the use of these agents in clinical and veterinary medicine.

Using the genes present in the VirulenceFinder database (76 genes plus variants), we found limited variation in gene content among the isolates. Differences were observed mostly among the atypical isolates because of the carriage of different plasmids. For example, ST76 SF E. coli O157 did not carry *espP*, *katP*, and *toxB*, which is consistent with the carriage of the pSFO157 plasmid (46). This strain also did not carry *espF*, *iha*, and *tccP* but harbored *cdtB*. Eklund et al. (47) found most SF E. coli strains to possess the gene cluster (*cdtV*) encoding the cytolethal distending toxin family, which includes *cdtB*. Among the NSF E. coli O157 isolates, we found *cdtB* exclusively within sublineage Ib, so it may be suitable for use as a marker for this sublineage.

Although the virulence genes were detected with a high degree of accuracy, some discordant results were observed, mostly related to the limitation of short sequence read assembly to resolve repeat regions and, in some cases, low read depth resulting in poor assemblies. The sensitivity of *in* silico detection software, such as VirulenceFinder, depends upon the use of high-quality assemblies, and assembly efficiency is dependent on read depth across the whole genome. Jünemann et al. (48) compared benchtop sequencers to show that the optimal coverage (based on N50 values) with sequencing data generated by the PGM and MiSeq was ∼40- and 75-fold, respectively, while Desai et al. (49) reported that the coverage required to produce a good assembly was dependent on the assembly software used; however, 50× was optimal for most of the widely used software (e.g., Velvet, SOAPdenovo, and AbySS) to assemble an E. coli genome with Illumina reads. Other gene-finding software (e.g., Ridom SeqSphere) suggests a minimum coverage of ≥50× for PGM data and ≥75× for MiSeq data (Ridom GmbH, Münster, Germany). In this study, the average read depth ranged from 23 to 106 (see Table S1 in the supplemental material); therefore, in some cases, optimal coverage was not obtained, explaining, in part, the failure of VirulenceFinder to detect some genes. However, a gene-finding method that does not consider each contig separately may make better use of the available sequencing information.

The VirulenceFinder database also contained the *stx* genes as individual subunit sequences rather than holotoxins; however, the combination of both subunit sequences is essential for accurate differentiation of the subtypes, explaining why, in some cases, two subunits lying adjacent to each other were reported as different *stx* subtypes. It is essential that the *stx* genes can be accurately detected and characterized, as they play an essential role in the development of disease and some studies (38, 39, 40) have found that certain subtypes (e.g., *stx*_2a_) have been associated with increased pathogenicity. WGS has the advantage of being able to detect multiple *stx* genes of the same subtype, which is not possible by PCR. For example, in one strain, we detected two different *stx*_2a_ gene variants. Recently, Ashton et al. (50) used a combined mapping and *de* *novo* approach to detect multiple genes of the same *stx* subtype in ∼15% of the isolates tested. In this study, we may have underestimated the number of isolates with multiple genes of the same subtype, as the bioinformatics approach used may not have detected these. The significance of the presence of multiple genes of the same *stx* subtype is unknown and needs further investigation.

It is currently not possible to determine MLVA types from WGS data because of short read lengths; however, with advances in technology such as single-molecule real-time sequencing (e.g., PacBio) (51) and nanopore sequencing (52), which provide longer read lengths capable of spanning tandem repeat regions, this information will be captured and will be available to help define the evolutionary relationships of isolates. The molecular basis of phage typing is also not known, so it is currently not possible to extract PT from WGS data. MLST types were successfully determined *in* silico. Although the 15-locus scheme provides a low level of resolution, there are various softwares (Applied Maths, Ridom SeqSphere, BIGSdb) available for whole-genome MLST, enabling the analysis of thousands of genes. It is thought that whole-genome MLST will be easier to standardize than core SNP analysis and enable a hierarchical approach to be used (53); however, the method requires allele curation and may be less discriminatory.

The development of improved software to overcome the limitations of short read assembly is required to realize the potential of WGS for in silico data analysis. Precise knowledge and understanding of the genome contents of the strains causing clinical infections should enable the identification of risk factors for improved patient management. We have shown that Ic3 strains were significantly associated with severe disease; however, contrary to recent work (38), we did not find carriage of *stx*_2a_ to be essential for the development of HUS. Further in-depth analysis of these Ic3 strains is required to determine features likely to be important in the development of severe disease.

Overall, we have demonstrated that WGS offers the potential to streamline reference laboratory processes by the use of a single diagnostic tool to generate the information required to support the clinical management of cases and public health investigations and interventions to control disease spread. It has the potential to transform the way we assess the relatedness of strains and the risk of development of severe complications. However, issues relating to ease of performance and standardization, as well as information technology infrastructure and data storage, need to be addressed before it is introduced routinely.

## Supplementary Material

Supplemental material
