# Supplementary material for: Utility of Whole-Genome Sequencing of Escherichia coli O157 for Outbreak Detection and Epidemiological Surveillance
Source: J Clin Microbiol. 2015 Oct 16;53(11):3565–73. doi: 10.1128/JCM.01066-15 (PMC4609728; doi:10.1128/JCM.01066-15)
Supplement: Supplemental material [file supp_53_11_3565__index.html]

Utility of Whole-Genome Sequencing of Escherichia coli O157 for Outbreak Detection and Epidemiological Surveillance — Supplemental material 

# Utility of Whole-Genome Sequencing of Escherichia coli O157 for Outbreak Detection and Epidemiological Surveillance

## Supplemental material

- Supplemental file 1 -

  Fig. S1 (Plots of number of variable sites in 10,000-bp windows across the *E. coli* O157 core genome alignment), S2 (Clustering of O157 sequences), S3 (O157 RAxML tree with recombinant XH18570E and XH22083W sequences included), and S4 (Minimum spanning tree of MLVA data for the 105 *E. coli* O157 isolates)

  PDF, 631K
- Supplemental file 2 -

  Table S1 (Isolate information and full set of genotypic and phenotypic results)

  XLS, 70K
- Supplemental file 3 -

  Table S2 (SNP distances between all pairs of isolates)

  XLSX, 107K
