# Supplementary material for: Utility of Whole-Genome Sequencing of Escherichia coli O157 for Outbreak Detection and Epidemiological Surveillance
Source: J Clin Microbiol. 2015 Oct 16;53(11):3565–73. doi: 10.1128/JCM.01066-15 (PMC4609728; doi:10.1128/JCM.01066-15)
Supplement: Supplemental material [file JCM.01066-15_zjm999094590so1.pdf]

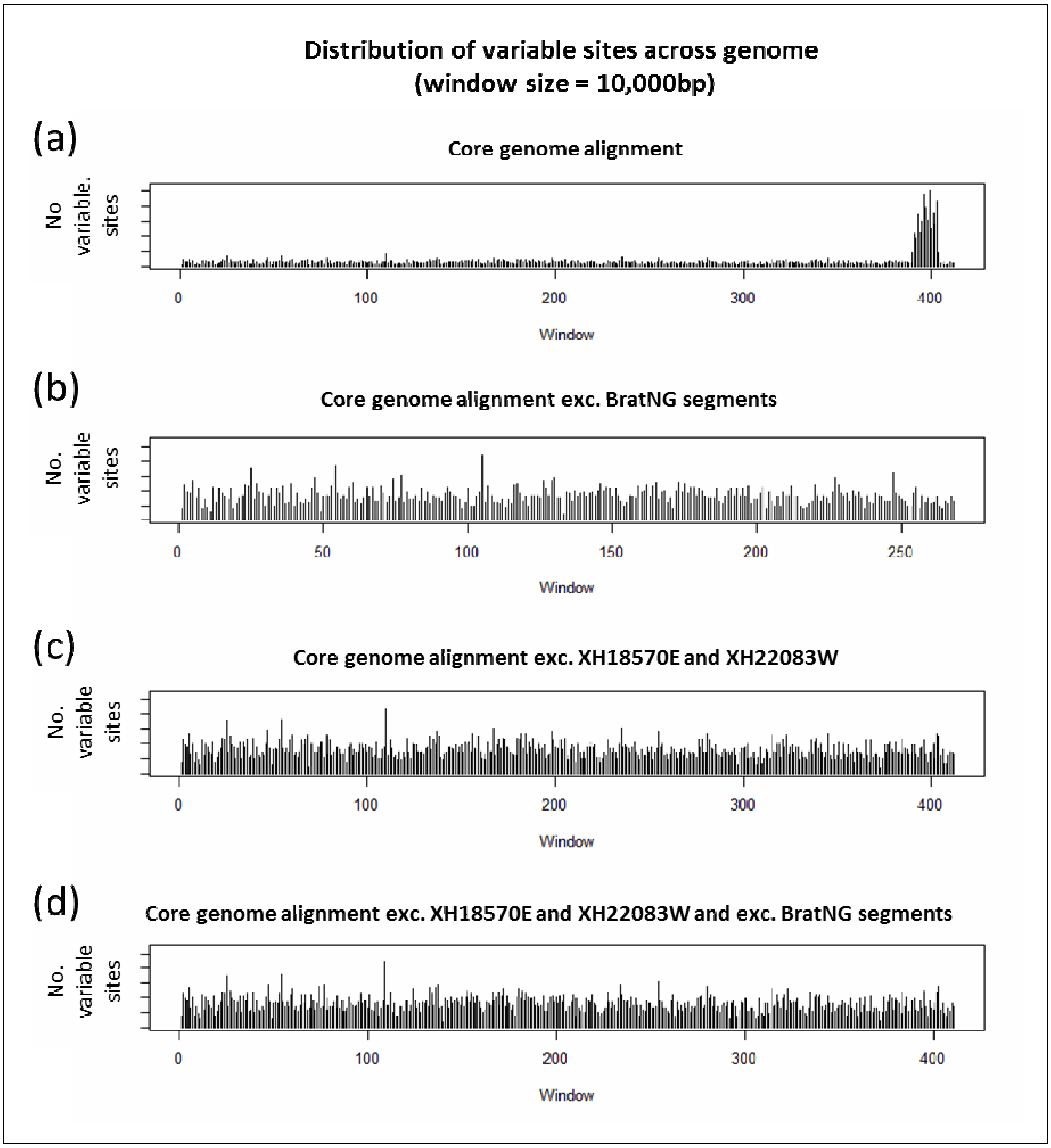

Figure S1: Plots of number of variable sites in 10,000bp windows across the E. coli O157 core genome alignment. (a) Plot for the initial core genome alignment, with all strains included and recombination unaccounted for; (b) plot for the core genome alignment, with all strains included and sites at which recombination was detected by the BratNextGen software removed from the alignment; (c) plot for the core genome alignment with recombinant strains XH18570E and XH22083W removed; (d) plot for the core genome alignment with strains XH18570E and XH22083W excluded, followed by recombination detection and subsequent removal of sites at which recombination was detected.

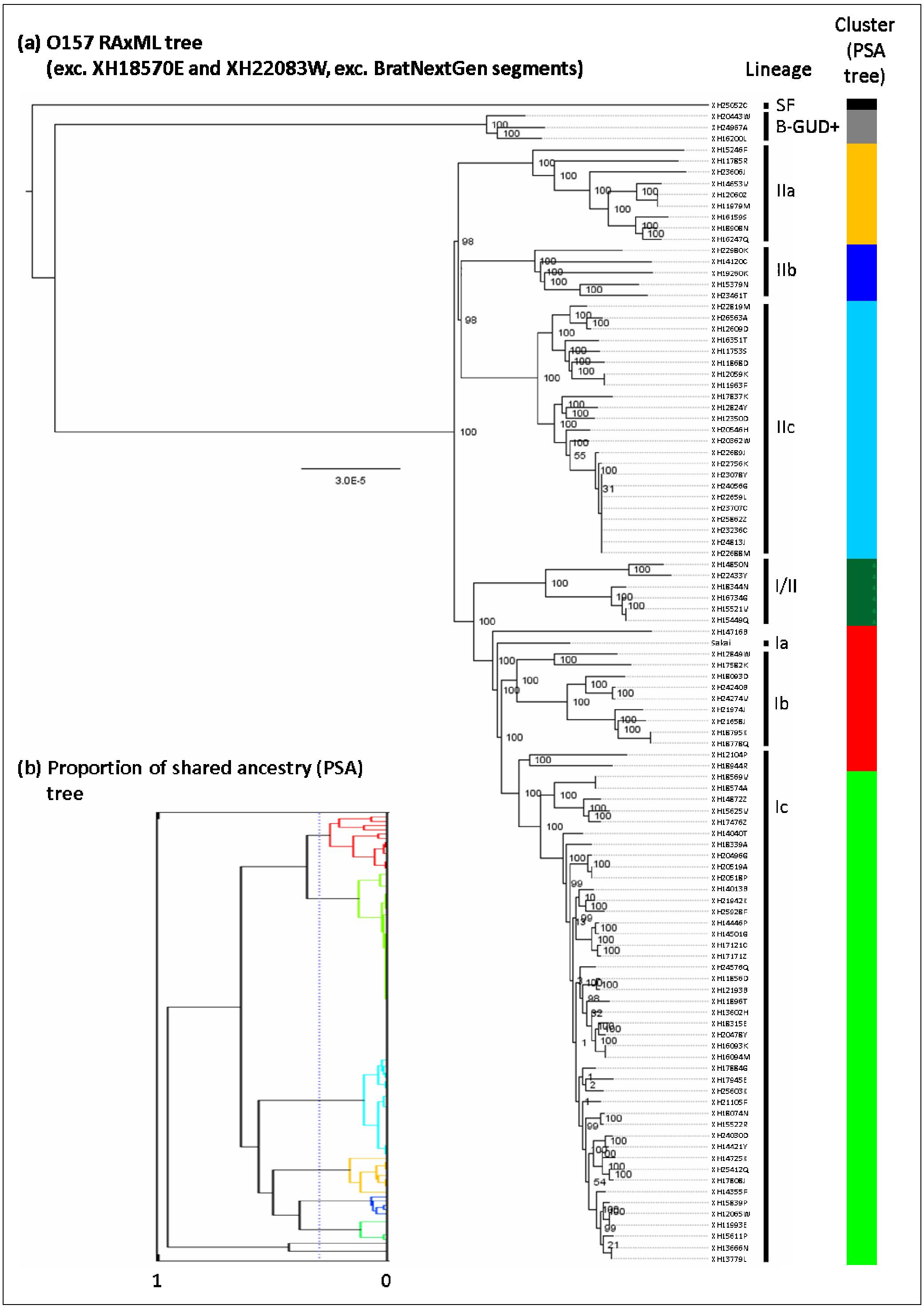

Figure S2: Clustering of O157 sequences. (a) Classification of *E. coli* O157 sequences into clusters defined from the proportion of shared ancestry (PSA) tree during BratNextGen analysis. Cluster membership is represented by coloured blocks alongside the maximum likelihood phylogeny (same phylogeny as Figure 1). A strong concordance was observed between the inferred clusters and the *E. coli* O157 lineages defined by Dallman *et al.*, which are also depicted alongside the maximum likelihood tree. (b) Clusters calculated in BratNextGen based on the proportion of shared ancestry with a cutoff (indicated by a dotted line) which divided the isolates into 8 clusters.

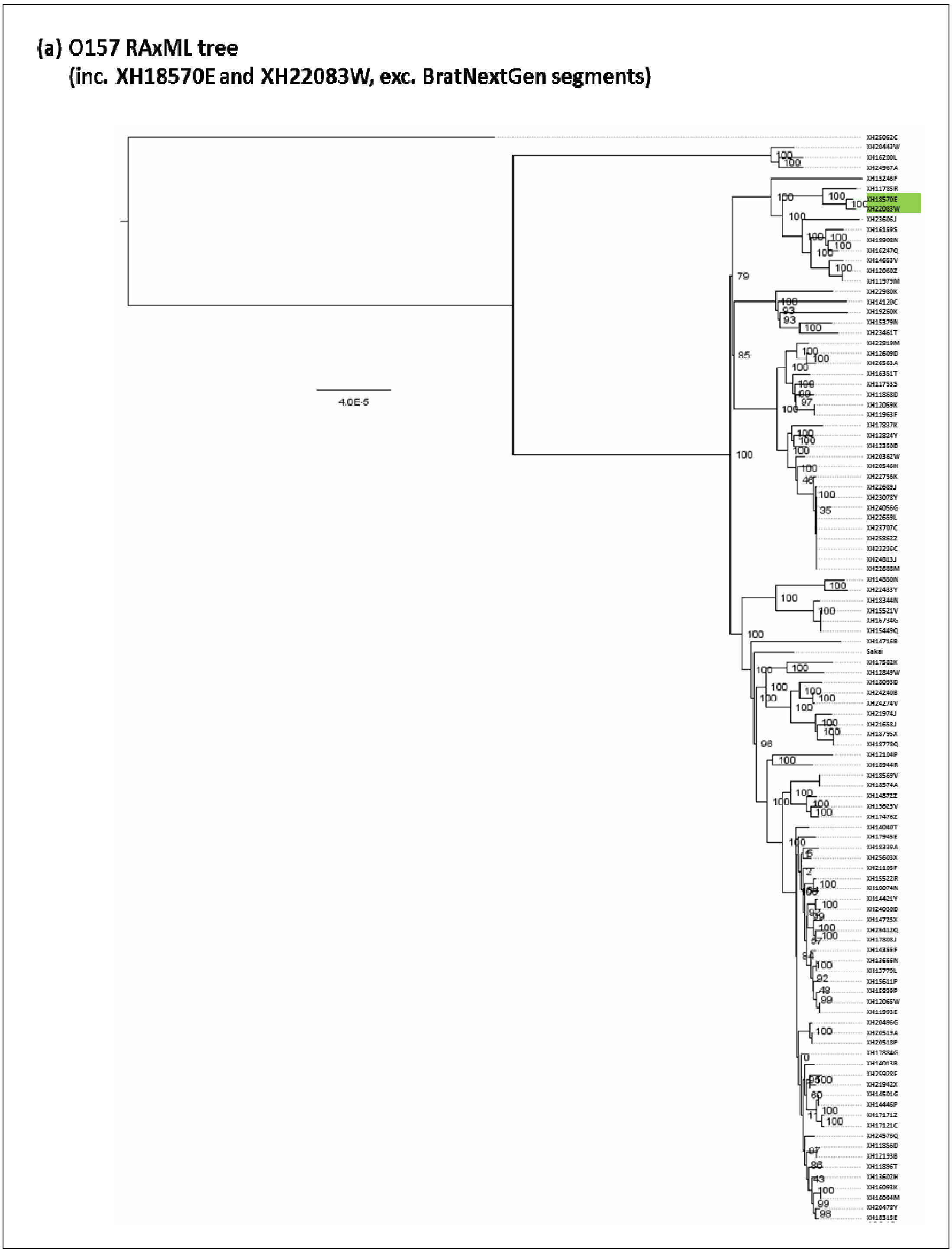

Figure S3: O157 RAxML tree with recombinant sequences XH18570E and XH22083W included. A maximum likelihood tree was constructed in RAxML from a core genome alignment for the *E. coli* O157 sequences, with recombinant sequences XH18570E and XH22083W included but with recombinant regions of the genome (detected by BratNextGen) removed. The phylogenetic analysis was run using a general time reversible model of nucleotide substitution and gamma distributed rate heterogeneity across sites, and 1000 bootstrap replicates were conducted. Bootstrap values are displayed as percentages, and branch lengths are in units of substitutions per site. The recombinant sequences XH18570E and XH22083W are highlighted in green.

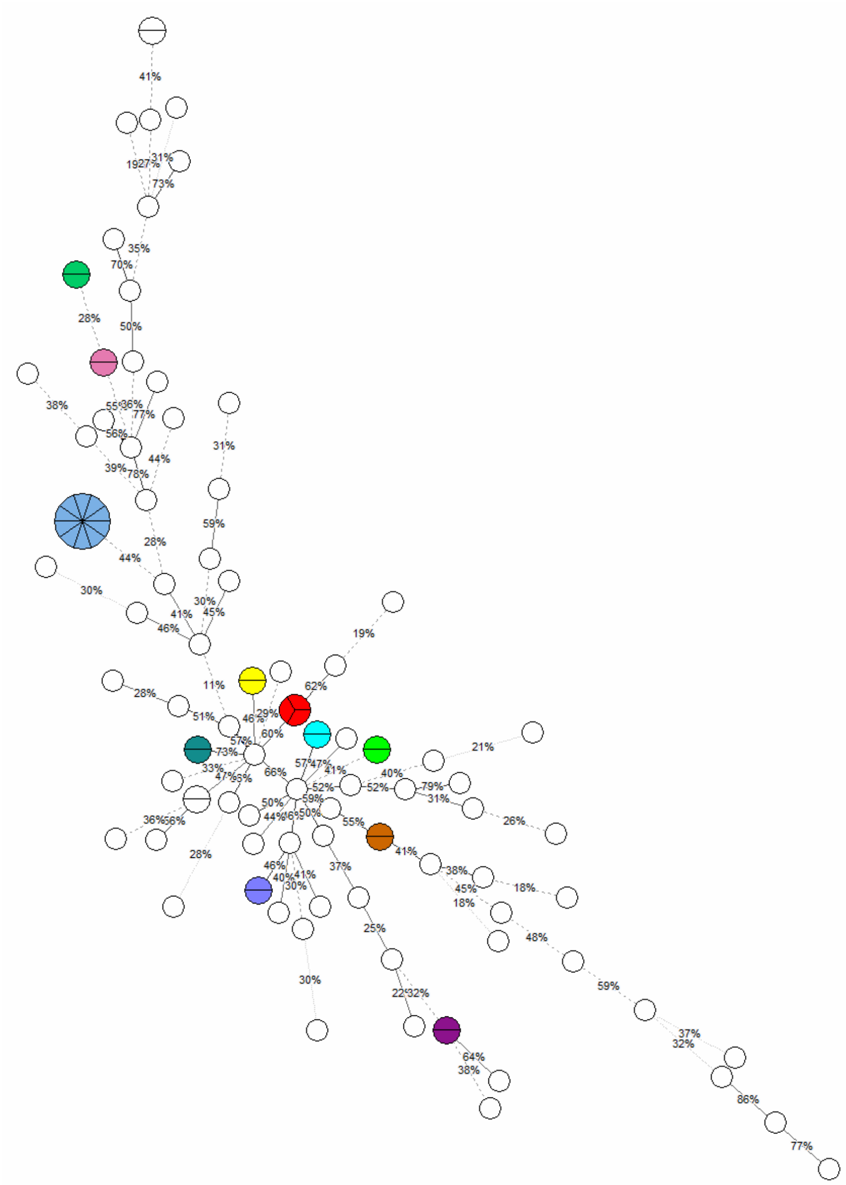

Figure S4: Minimum spanning tree of MLVA data for the 105 *E. coli* O157 isolates. The size of the circle is proportional to the number of isolates that have a particular type. Known epidemiologically related isolates are shown in different colours. Bootstrap values are expressed as percentages.
